# Gut microbiome composition associates with corticosteroid treatment, morbidity, and senescence in Chinook salmon (*Oncorhynchus tshawytscha*)

**DOI:** 10.1038/s41598-023-29663-0

**Published:** 2023-02-13

**Authors:** Claire E. Couch, William T. Neal, Crystal L. Herron, Michael L. Kent, Carl B. Schreck, James T. Peterson

**Affiliations:** 1 Department of Fisheries, Wildlife, and Conservation Sciences, Oregon State University, Corvallis, OR USA; 2 Department of Microbiology, Oregon State University, Corvallis, OR USA; 3 Carlson College of Veterinary Medicine, Oregon State University, Corvallis, OR USA; 4 U.S. Geological Survey Oregon Cooperative Fish and Wildlife Research Unit, Corvallis, OR USA

**Keywords:** Ecology, Microbiology, Physiology, Zoology

## Abstract

Pacific salmon experience prolonged elevation in corticosteroid hormones during important life history events including migration, reproduction, and senescence. These periods of elevated corticosteroids correspond with changes to immunity and energy metabolism; therefore, fish may be particularly vulnerable to mortality at these times. Recent studies found that stress-induced cortisol release associated with microbial community shifts in salmonids, raising the question of how longer-term corticosteroid dynamics that accompany life history transitions affect salmonid microbiomes. In this work, we experimentally evaluated the relationships between gut microbiome composition, chronically elevated corticosteroids, and mortality in juvenile Chinook salmon (*Oncorhynchus tshawytscha*). We found that treatment with slow-release implants of the corticosteroids cortisol or dexamethasone resulted in changes to the gut microbiome. Morbidity was also associated with microbiome composition, suggesting that the gut microbiome reflects individual differences in susceptibility to opportunistic pathogens. Additionally, we analyzed a small number of samples from adult fish at various stages of senescence. Results from these samples suggest that microbiome composition associated with gut integrity, and that the microbial communities of corticosteroid treated juveniles shift in composition toward those of senescent adults. Overall, findings from this work indicate that the gut microbiome correlates with mortality risk during periods of chronic corticosteroid elevation.

## Introduction

Corticosteroid hormones, particularly glucocorticoids, are critical regulators of many aspects of vertebrate physiology, including metabolism^1^, development^2^, and immunity^3^, in addition to playing a central role in the stress response^4^. Glucocorticoid dysregulation due to chronic stress, inflammatory disease, or other factors^5,6^, may disrupt these physiological processes, resulting in disease susceptibility^7^, reduced growth^8^, and reproductive failure^9^. In many species, resting glucocorticoid levels and the scope of the glucocorticoid stress response naturally fluctuate across the lifespan^10,11^. A dramatic example of this intra-individual variation is the pronounced elevation of glucocorticoids that accompanies the programmed senescence and death of semelparous vertebrates^12,13^. In these species, cortisol rises to extreme levels leading up to reproduction, perhaps to facilitate the intense energetic demands associated with reproduction. This extreme rise in cortisol may be ultimately responsible for their inevitable post-reproductive death. Thus, while long-term elevation of glucocorticoids may facilitate important life history events such as reproduction, it may also incur a tradeoff by increasing mortality risk, especially in populations exposed to significant external stressors. While most glucocorticoid studies focus on the acute stress response, identifying the causes and consequences of chronic elevation of glucocorticoids is important for understanding the life history and ecology of vertebrate populations.

The gut microbiome is a potentially important modulator of corticosteroid dynamics in vertebrates. Recent studies indicate a bidirectional relationship between the gut microbiome and glucocorticoids in the context of acute stress. Direct administration or stressor-induced elevation of glucocorticoids can alter the diversity and composition of the gut microbiome in mammals, birds, and fishes^14–16^, and in turn, the gut microbiome can contribute to steroid metabolism^17^ and mediation of the behavioral and physiological effects of glucocorticoids on the host. In salmonid fishes, acute stress-induced cortisol elevation correlates with altered community richness and composition of the skin and fecal microbiome, and may promote the proliferation of opportunistic pathogens^16,18^. The effect of glucocorticoids on the gut microbiome could be mediated by the host immune system, as disease and immune status have also been linked with dysbiosis of the gut^19,20^. Conversely, the microbiome appears to modulate the effects of glucocorticoids on the host, though the mechanisms of this relationship are not yet well understood^21^. For example, prebiotic modulation of the gut microbiome reduces the waking cortisol response and alters emotional bias in humans^22^. Germ-free rats experience higher glucocorticoid levels and anxiety behaviors than those with normal microbiota^23^. Similarly, in zebrafish, antibiotic treatment to reduce native bacteria resulted in increased anxiety-like behaviors^24^.

Pacific salmon (*Oncorhynchus* sp.) are a genus of fishes that are primarily anadromous, meaning that they migrate from saltwater to freshwater to spawn, and semelparous, meaning that they die after a single reproductive event. In members of this genus, cortisol (the primary glucocorticoid in fishes) rises during major life history events including the transition of young fish from freshwater to saltwater (smolting)^25^, adult migration^26^, sexual maturation^27^, and finally, senescence^28^. During life stages with chronically elevated cortisol, Pacific salmon are particularly susceptible to external stressors such as disease, thermal stress, and physical exertion^29^. Therefore, understanding the causes and consequences of chronic cortisol elevation is important for predicting susceptibility to external stressors. Previous studies in other salmonid species have identified correlations between cortisol and microbiome variation following acute stressors^16,18^; therefore the gut microbiome is also likely to be affected by chronic cortisol elevation. Both the microbiome^30,31^ and chronic cortisol elevation^29^ have been linked to immune suppression, so it is likely that microbiome-immune crosstalk mediates pathogen susceptibility^32^. Cortisol is known to affect inflammatory pathways in salmonids^33,34^, and it may be that the microbiome is involved in the relationships between life history, cortisol, and immunity these fishes.

In this study, we explored the effects of chronic corticosteroid elevation on the gut microbiomes of Chinook salmon (*O. tshawytscha*). To isolate the effects of corticosteroids versus other life history-associated physiological processes and environmental variables, we experimentally administered slow-release corticosteroid implants to pre-smolt juveniles under controlled environmental conditions. Additionally, we compared the gut microbiota of experimental juveniles with opportunistically collected samples from adult fish at different stages of senescence. We hypothesized that (i) chronic glucocorticoid elevation would cause gut dysbiosis due to changes in immune function, resulting in increases of potentially pathogenic taxa^18^. We also hypothesized that (ii) degree of dysbiosis would reflect individual ability to cope with elevated corticosteroids, and hence the degree of dysbiosis would associate with host morbidity.

## Methods

### Experimental methods and sample collection

All animal husbandry methods and experimental procedures were performed in accordance with relevant guidelines and regulations and were approved by Oregon State University Institutional Animal Care and Use Committee (ACUP #2020-0119). We obtained 300 juvenile Chinook salmon weighing approximately 20 g each from South Santiam Hatchery in Sweet Home, Oregon, USA (44°24′57.5″N 122°40′32.8″W) on August 31, 2020. Fish were held at the Fish Performance and Genetics Laboratory (FPGL) in Corvallis, Oregon (44°57′62.5″N, 123°24′11.6″W) in gravel filtered well water at a density of approximately 2 kg fish/cubic meter. After 4 weeks, 150 of the fish were transported to the adjacent Aquatic Animal Health Laboratory (AAHL) where they were held in tanks with a mixture of UV-sterilized water (2 L/min) from the same groundwater source as the FPGL water, and untreated water pumped directly from the Willamette River (1 L/min). The purpose of the untreated river water was to facilitate exposure to a natural suite of microbes similar to what would be encountered by fish in the wild and determine whether the effects of corticosteroid treatment on the microbiome were consistent between water sources. Water temperature at FPGL was held constant at 12.5 °C and fluctuated between 10.8–13.4 at AAHL due to changing temperature of river water input. Throughout the study, fish were fed 1.2% body weight per day of Bio-OR^®^ BioClark’s Fry diet.

Fish were divided into groups of 50 for a total of six tanks (three tanks in well water at FPGL and three tanks in a mixture of well water and untreated river water at AAHL) and allowed to acclimate for an additional four weeks, then PIT tagged and administered with corticosteroid implants in late October. Fish were anesthetized prior to PIT tagging and all subsequent handling events by immersion in 50 mg/L tricaine methanesulfonate (MS-222) buffered to neutrality with sodium bicarbonate.

Slow-release corticosteroid implants were prepared by dissolving 10 mg of either dexamethasone or cortisol in 200 μL of sterile Crisco^®^ vegetable shortening. Warmed, liquid phase implants were administered to fish by intraperitoneally injecting into the abdominal cavity, ventral to the pyloric cecae, using 21-gauge needles. We administered cortisol, dexamethasone, or control (vegetable shortening only) implants to each fish (16 or 17 of each treatment group in each tank). At three weeks post treatment, we anesthetized all fish and collected gut swabs by gently inserting a small, sterile 2 mm diameter cotton swab approximately 1 cm into the anal vent. Half the fish from each treatment group in each tank were then randomly selected for euthanasia by submersion in 250 mg/L MS-222, and blood was collected with sodium heparinized 21-gauge needles and centrifuged for 5 min at 10,000*g* to obtain plasma immediately after euthanasia. Swabs, plasma samples, and carcasses were immediately placed in ice for transport to the laboratory. Swabs and plasma samples were frozen at − 80 °C within four hours of collection. Visceral organs were collected from carcasses and preserved in Dietrich’s fixative for histology, and a 10 mm section of the most distal portion of the intestine was frozen for microbiome analysis in addition to the gut swabs. At seven weeks post-treatment, the same procedure was conducted on all remaining fish (terminal samples). Any fish that became moribund (generally ataxic accompanied by loss of buoyancy control and/or increased respiration rate) between the first and second sampling were humanely euthanized using 250 mg/L MS-222 and are referred to as mortalities. Other organs in addition to the intestine were examined by histology to provide a broad assessment of pathologic changes associated with morbidity. Gill, intestine, kidney, liver, spleen and heart were collected from three surviving and ten moribund dexamethasone-treated fish, four surviving and two moribund cortisol-treated fish, and seventeen surviving control fish and preserved in Dietrich’s solution for histology. Internal organs and gills were processed into histologic slides and stained with hematoxylin and eosin by the Oregon Veterinary Diagnostic Laboratory (Corvallis, Oregon, USA).

### Adult fish samples

In addition to the juvenile experimental fish, we collected analogous anal vent swabs from adult Chinook salmon carcasses immediately after fish were artificially spawned for propagation at Willamette Hatchery in Oakridge, Oregon, USA (43°73′84.9″N, − 122°43′77.2″W) in September of 2020. Samples were immediately placed on ice and were frozen at − 80 within 3 h of collection. To evaluate senescence via histology, we preserved lower intestine and pyloric caeca samples in Dietrich’s fixative for histology and evaluated the integrity of the epithelium by measuring the percent of intact gut epithelium remaining^35^. Scoring of gut samples was conducted by an experienced fish pathologist with forty years of experience conducting histological analysis of salmonids. We previously demonstrated that loss of integrity of the intestinal epithelium correlates closely with the timing of senescence and mortality in adult freshwater phase Chinook salmon^35,36^. Therefore, to explore potential differences in gut microbiota between adult fish at early versus late stages of gut senescence, we used epithelial integrity scores to select four adult males with extremely degraded gut epithelia (“senescent”) and three adult males with mostly intact gut epithelia (“pre-senescent”) for microbiome sequencing. Fish designated as senescent had 5–10% epithelial integrity, and fish designated as pre-senescent had 70–80% epithelial integrity.

### Plasma cortisol

Cortisol concentration was determined via radioimmunoassay using methods described by Redding et al.^37^. Briefly, 10 µL of whole plasma was heat denatured to liberate any endogenously bound cortisol in the sample. The endogenous cortisol was allowed to compete with 1,2,6,7 ^3^H Cortisol (Perkins Elmer NET 39600) for a known amount of added cortisol antibody (Fitzgerald Industries International Ind. Cat No. 20-CR50). Samples were further diluted in Economical Biodegradable Counting Cocktail (Econo-Safe) and counts per minute were determined over 5 min using a Beckman Coulter LS 6500 Multi-Purpose Scintillation Counter. Samples were analyzed in duplicate and average cortisol concentration per sample was determined.

### Sample processing and sequencing

We analyzed microbiome samples from 90 fish. Juvenile samples were from one tank at the FPGL (well water only) and one tank at the AAHL (well water and river water). These samples included 64 juveniles sampled at three weeks post treatment, 21 of which became moribund prior to the end of the study, and 19 juveniles sampled at seven weeks post treatment (Table S1), in addition to the seven adult fish collected at Willamette Hatchery.

DNA extraction, library preparation, amplicon sequencing, initial quality control, and demultiplexing of swabs and gut tissue sections were conducted by the Oregon State University Center for Qualitative Life Science according to the Earth Microbiome Protocol^38–40^. Briefly, DNA extraction was conducted using the Qiagen MagAttract PowerSoil DNA kit (catalogue number 27100-4-EP) according to the Earth Microbiome Protocol^38,41^. This protocol has successfully been used to extract DNA from fish gut samples in previous studies (e.g.^42^). The V4 region of the 16S gene was amplified and barcoded with Earth Microbiome Primers 515F^43^ and 806R^44^. A single Illumina MiSeq lane (v.3 chemistry) was used to generate paired-end 2 × 300 bp reads for all samples.

All subsequent data processing and statistical analyses were conducted in R version 4.1.2^45^. Demultiplexed sequences were processed through DADA2 version 1.22.0^46^ to identify amplicon sequence variants (ASVs), trim adapter sequences, remove chimeras, and assign taxonomy based on the SILVA database version 132^47^. The following DADA2 trimming parameters were used: truncLen = c(240, 160), maxN = 0, maxEE = c(2,2), truncQ = 2, rm.phix = FALSE. Default parameters were used for estimating error parameters using learnErrors(), and chimeras were removed using removeBimeraDenova (method = “consensus”). Sequences were aligned using DECIPHER version 2.22.0^48^. A phylogenetic tree was constructed de novo using phangorn version 2.8.1^49^. A neighbor joining tree was constructed and used as a starting point for a generalized time reversible tree which was constructed using the following optimization parameters: optInv = TRUE, optGamma = TRUE, rearrangement = “stochastic”, control = pml.control.

Following ASV identification, taxonomic annotation, and construction of the phylogenetic tree, ASVs from the juvenile experiment were rarefied to the minimum sequencing depth, and ASVs that were not classified to the phylum level were removed prior to subsequent analyses. For comparisons between adults and juveniles, all ASVs were first rarefied to the minimum sequencing depth for adults.

### Statistical analysis

Cortisol concentrations resulting from the plasma cortisol radioimmunoassay were log-transformed and compared with time and treatment using a linear mixed-effects model with tank as a random intercept. A full model with an interaction term for time and treatment was fitted and compared to progressively reduced models including slopes for time and treatment, time only, treatment only, and intercept only using chi-squared tests.

Log richness and Shannon diversity^50^ for juvenile fish microbiomes were calculated from rarefied ASV counts. Linear mixed effects models with treatment as a fixed effect and time point, tank as random effects, and Shannon diversity or log richness as response variables were used to assess the effects of corticosteroid treatments on alpha diversity.

Nonmetric multidimensional scaling^51^ was performed on weighted unifrac distances using the metaMDS function in vegan^52^, with a minimum of 20 and a maximum of 1000 random starts. Permutational multivariate analysis of variance was performed on weighted unifrac distances to simultaneously assess the marginal effects of treatment, tank, time, and mortality using the adonis2 function in vegan. Pairwise permutational analysis of variance was used to directly compare treatment groups at the first time point (3 weeks post treatment) while stratifying by tank using the pairwise.adonis2 function in the pairwiseAdonis package^53^ using 999 permutations.

Compound Poisson generalized linear mixed models implemented in the cpglmm function of the cplm package^54^ version 0.7.9 were used to assess the effects of corticosteroid treatment on individual bacterial genera. Based on results from the plasma cortisol assays, we included only the 64 samples collected after three weeks in the treatment models. Because we were interested in identifying tractable biomarkers of corticosteroid treatment, models were only constructed for genera that were present in more than 10% of samples from the first sample point, resulting in a total of 212 genera. For each taxon, we constructed a full model (Eq. 1) and a reduced model (Eq. 2) and conducted an ANOVA F-test comparing the two models to ascertain whether including treatment resulted in improved model fit. False discovery rate adjusted p-values (q) resulting from the ANOVA F-test were used to identify taxa that associated significantly with treatment using a cutoff of q < 0.1. For genera that met this criterion, a post hoc model was generated to assess the effect of cortisol and dexamethasone treatment separately relative to mean abundance of that taxon (Eq. 3). This model was compared to reduced Eqs. (4) and (5) with an ANOVA F-test to assess whether including dexamethasone or cortisol significantly improved model fit (q < 0.1). For significant genera (q < 0.1), the parameter estimate for the relevant treatment from Eq. (3) was recorded. To assess the relationship between taxa abundances and mortality, we constructed another set of compound Poisson generalized linear mixed models with mortality as a binary fixed effect and treatment and tank as random effects (Eq. 6). Each genus model was compared to a reduced random effects model (Eq. 7) using an ANOVA F-test, and significant genera (q < 0.1 and the mortality parameter estimate from Eq. (6) were recorded.

1 $${\text{Count }}\sim {\text{ Treatment }} + \, \left( {{1}|{\text{Tank}}} \right)$$

2 $${\text{Count }}\sim \left( {{1}|{\text{Tank}}} \right)$$

3 $${\text{Count }}\sim {\text{ Cortisol Treatment }} + {\text{ Dexamethasone Treatment }} + \, \left( {{1}|{\text{Tank}}} \right)$$

4 $${\text{Count }}\sim {\text{ Cortisol Treatment }} + \, \left( {{1}|{\text{Tank}}} \right)$$

5 $${\text{Count }}\sim {\text{ Dexamethasone Treatment }} + \, \left( {{1}|{\text{Tank}}} \right)$$

6 $${\text{Count }}\sim {\text{ Mortality }} + \, \left( {{1}|{\text{Tank}}} \right) \, + \, \left( {{1}|{\text{Treatment}}} \right)$$

7 $${\text{Count}}\sim \left( {{1}|{\text{Tank}}} \right) \, + \, \left( {{1}|{\text{Treatment}}} \right)$$

### Ethical approval

All animal husbandry methods and experimental procedures were performed in accordance with relevant guidelines and regulations and were approved by Oregon State University Institutional Animal Care and Use Committee (ACUP #2020-0119). This study was conducted in accordance with ARRIVE guidelines.

## Results

Based on chi-squared tests of model subsets, the best model for cortisol included only treatment as a fixed effect. Cortisol treatment was associated with an average increase of 2.07 ng/mL in plasma cortisol (p = 0.00168), whereas dexamethasone treatment was associated with a light but not significant decrease of 1.19 ng/mL (p = 0.52). Plasma cortisol decreased slightly but not significantly between three weeks and seven weeks post-treatment, suggesting a slight attenuation of treatment effects (Fig. S1).

Histological analysis revealed no detectable changes to intestinal tissues or other visceral organs in control or treated fish. However, the gills of fish treated with cortisol or dexamethasone consistently exhibited moderate to severe diffuse epithelial hyperplasia and necrotic changes, accompanied by bacterial gill disease evidenced by a mixture of surface bacteria and the protozoan flagellate *Ichthyobodo necator*. Gills from some treated fish also had bacterial colonies within gill tissues suggestive of *Aeromonas salmonicida* (Supplementary Fig. S2), but this specific infection was not verified with other methods.

**Figure 1:**
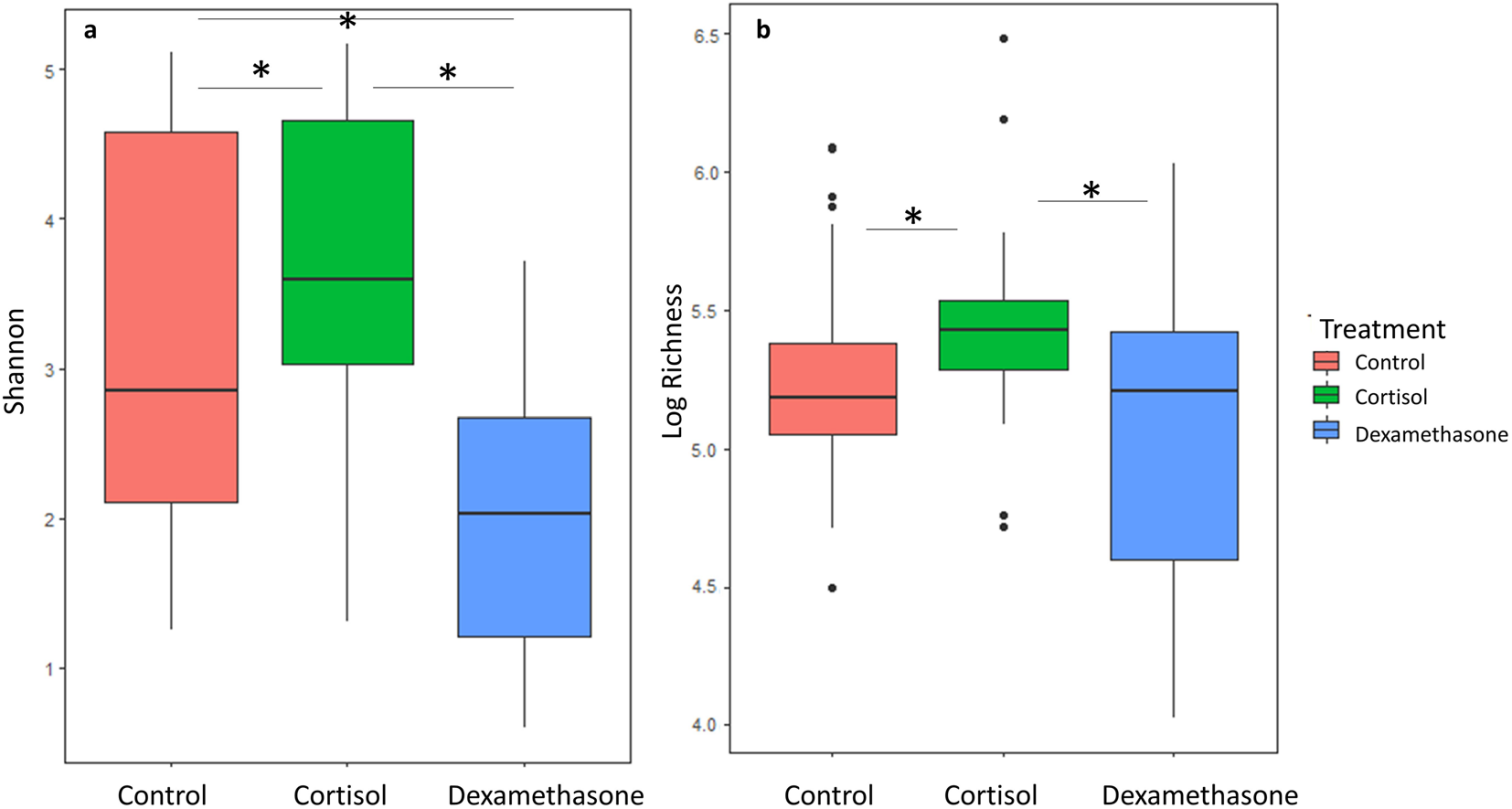
Boxplots of Shannon diversity (**a**) and log-transformed ASV richness (**b**) of juvenile gut microbiome samples from both time points (three and seven weeks post-treatment). The horizontal line bisecting the box indicates the median, the lower and upper hinges correspond to the first and third quartiles, and the upper and lower whiskers extend to the largest and smallest values no further than 1.5 IQR from the adjacent hinge. Significant differences between treatment groups are indicated with a line and an asterisk.

From the microbiome sequencing results obtained from the juvenile gut swabs, 9101 unique ASVs were identified from a total of 4,183,523 reads. Per-sample read depth ranged from 19,291 to 96,985 prior to subsampling to the minimum read depth and removing unclassified phyla. Shannon diversity was elevated in the cortisol treated fish relative to control fish (p = 0.009) and dexamethasone treated fish (p = 3.61e−06), and was reduced in dexamethasone treated fish relative to controls (p = 0.017). Richness was also elevated in cortisol treated fish relative to control (p = 0.034) and dexamethasone treated fish (0.025), and slightly but not significantly reduced in dexamethasone treated fish (p = 0.83) (Fig. 1). Overall richness and Shannon diversity did not differ significantly across facilities, but mean differences between corticosteroid treatment groups were greatest at the FPGL (Fig. S3). ASV richness in juveniles ranged from 56 to 655, with a mean of 211. In the adult samples, a total of 823 unique ASVs were identified from 250,098 reads. Per-sample read depth ranged from 8612 to 56,855 prior to subsampling and removing unclassified phyla. In adults, ASV richness ranged from 48 to 215 with a mean of 151.

**Figure 2:**
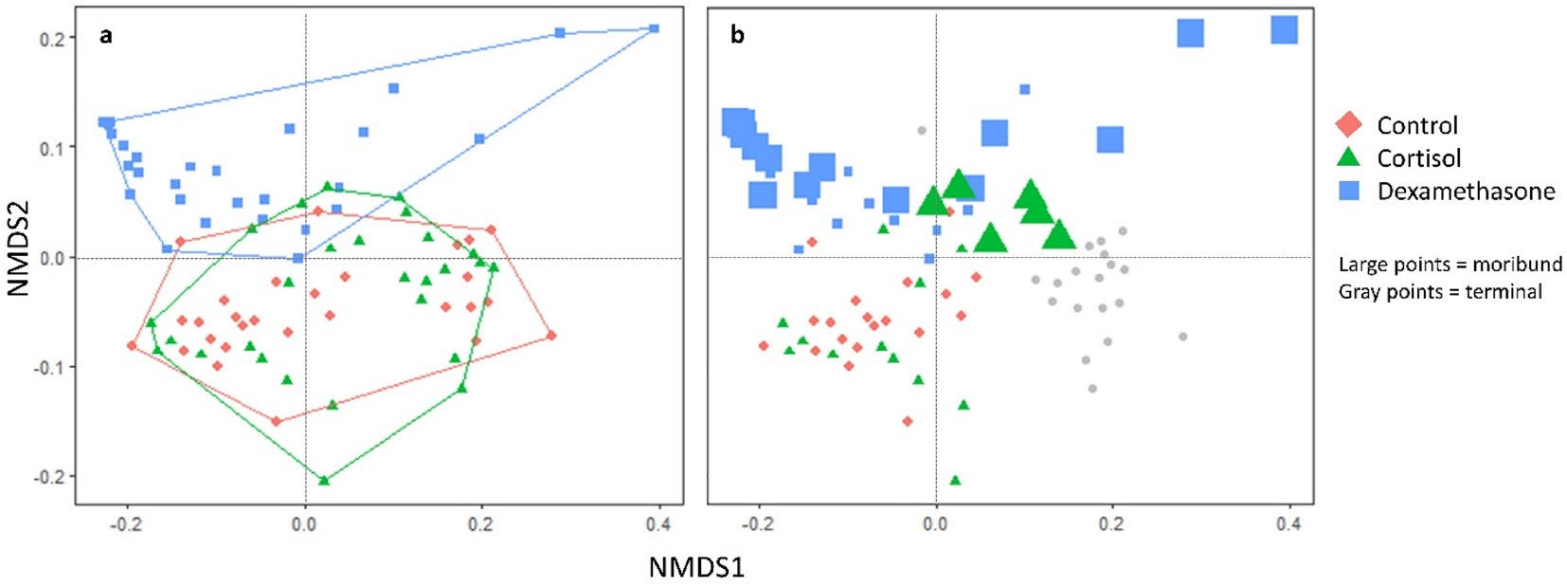
Nonmetric multidimensional scaling of weighted unifrac distances among juvenile gut microbiome samples. (**a**) Beta diversity differed significantly between all treatment groups based on PERMANOVA, while beta dispersion did not differ. (**b**) Beta diversity associated with subsequent mortality after controlling for treatment and tank. Grey points denote terminal samples.

**Figure 3:**
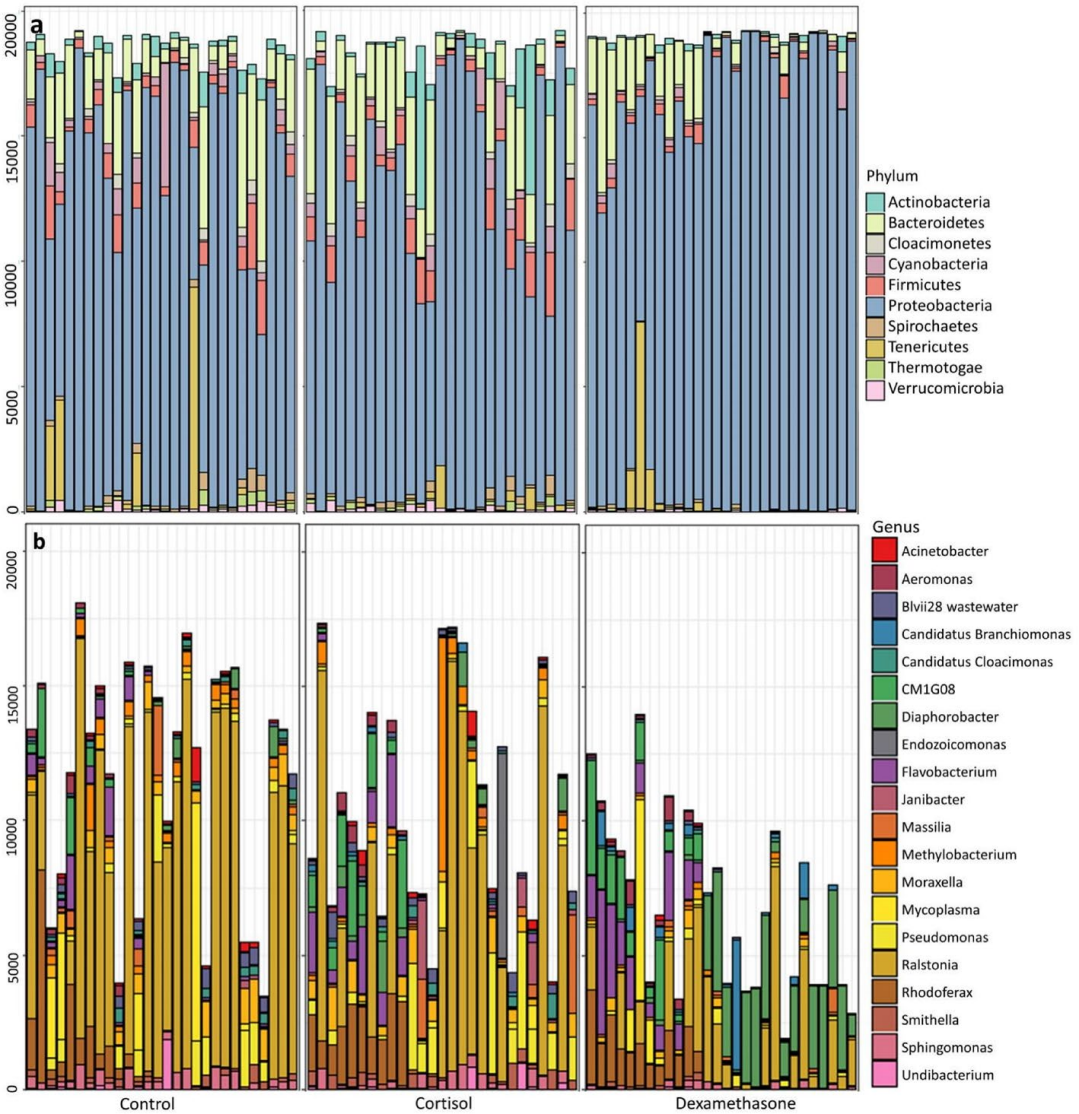
Rarefied abundances of (**a**) the top ten most abundant phyla and (**b**) the top 20 most abundant genera present juvenile Chinook salmon treated with vehicle-only control, cortisol, or dexamethasone implants.

A convergent NMDS solution for juvenile microbiome samples was found after 100 iterations, and the stress was 0.1. Visualization of the NMDS results revealed microbiome community shifts relative to treatment, time, and mortality (Fig. 2). PERMANOVA analysis found that treatment, tank, time point, and mortality in juveniles significantly associated with weighted unifrac distance (Table 1). Further analysis using pairwise PERMANOVA of treatment groups from the first time point demonstrated differences between cortisol versus control (R squared = 0.064, p = 0.04), dexamethasone versus control (R squared = 0.216, p = 0.001), and cortisol versus dexamethasone (R squared = 0.13, p = 0.001) treatments. Relative abundances of the top 10 phyla and the top 20 genera from the juvenile study are shown in Figs. 3, S4, S5, and S6.

**Table 1:** PERMANOVA results assessing the marginal effects of treatment, tank, time, and mortality on weighted unifrac distances of gut swabs from experimental juveniles.

Covariate	Degrees of freedom	Sum of squares	R squared	F statistic	P-value
Treatment	2	0.4269	0.07660	6.0030	0.001
Tank	1	0.5745	0.10307	16.1557	0.001
Time point	1	1.3571	0.24347	38.1629	0.001
Mortality	1	0.2024	0.03631	5.6919	0.002
Residual	77	2.7381	0.49125

We identified 54 genera associated with at least one of the two corticosteroid treatments based on Eqs. (1) and (2) and using data from only the first time point (q < 0.1, Supplementary File S1). Of these genera, 5 were positively associated with both cortisol and dexamethasone treatment and 6 were negatively associated with both treatments based on ANOVA F-tests comparing Eq. (3) to reduced models Eqs. (4) and (5) (p < 0.05). ANOVA F-tests comparing Eq. (6) with Eq. (7) identified two genera that positively associated with mortality and seven genera that negatively associated with mortality (q < 0.1, Table 2).

**Table 2:** Microbial genera associated with mortality.

Genus	Estimate	p-value	FDR adjusted p-value (q)
*Diaphorobacter*	0.848	0.004	0.094
*Fluviicola*	2.341	0.002	0.063
*Ralstonia*	− 2.465	< 0.001	< 0.001
*Methylobacterium*	− 2.091	< 0.001	< 0.001
*Sphingomonas*	− 1.675	< 0.001	< 0.001
*Meiothermus*	− 2.068	< 0.001	0.005
*Alishewanella*	− 3.955	< 0.001	< 0.001
*Corynebacterium_1*	− 1.630	0.002	0.063
*Microvirga*	− 28.56	0.003	0.078

**Figure 4:**
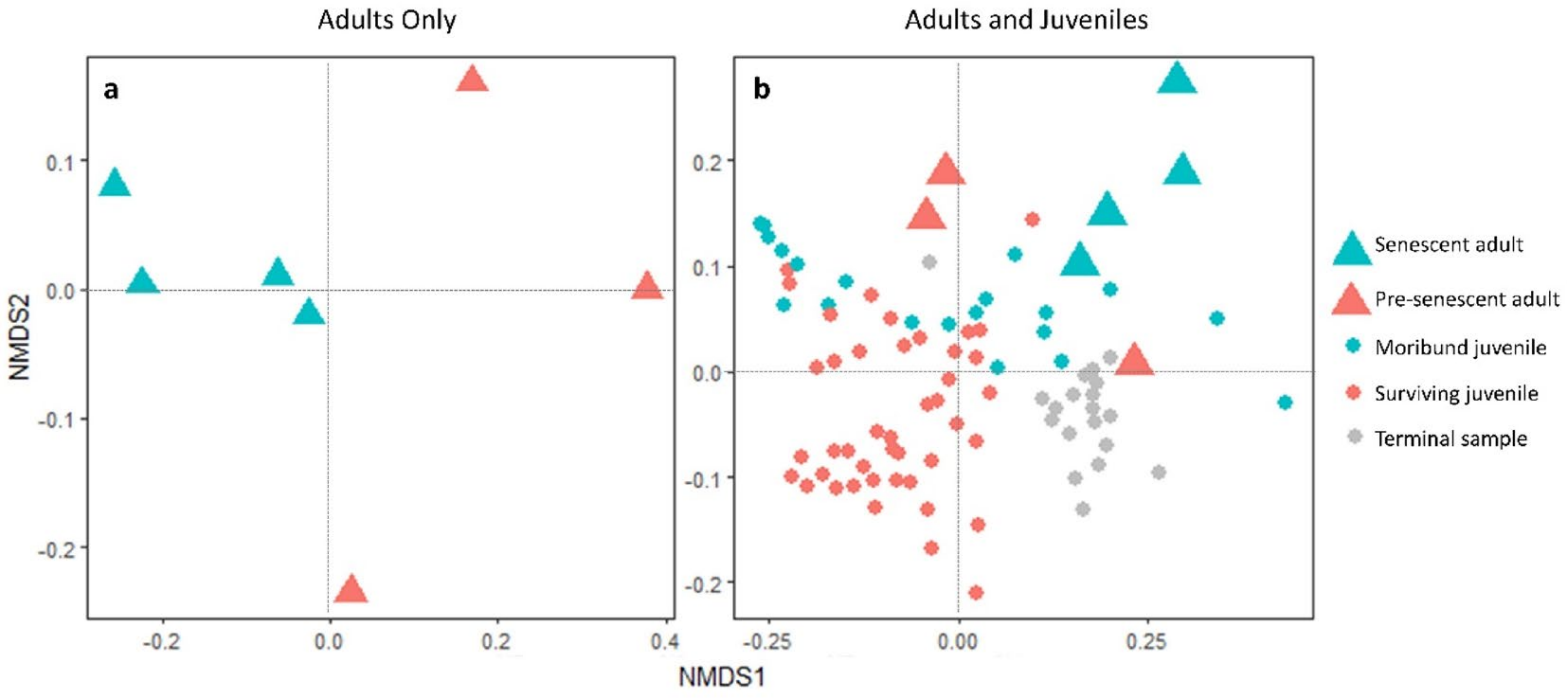
Nonmetric multidimensional scaling of weighted unifrac distances among adults and juveniles. (**a**) Beta diversity differed between early senescence and late senescence adults. (**b**) Adults in all stages of senescence appear more similar to moribund juveniles than surviving juveniles.

**Figure 5:**
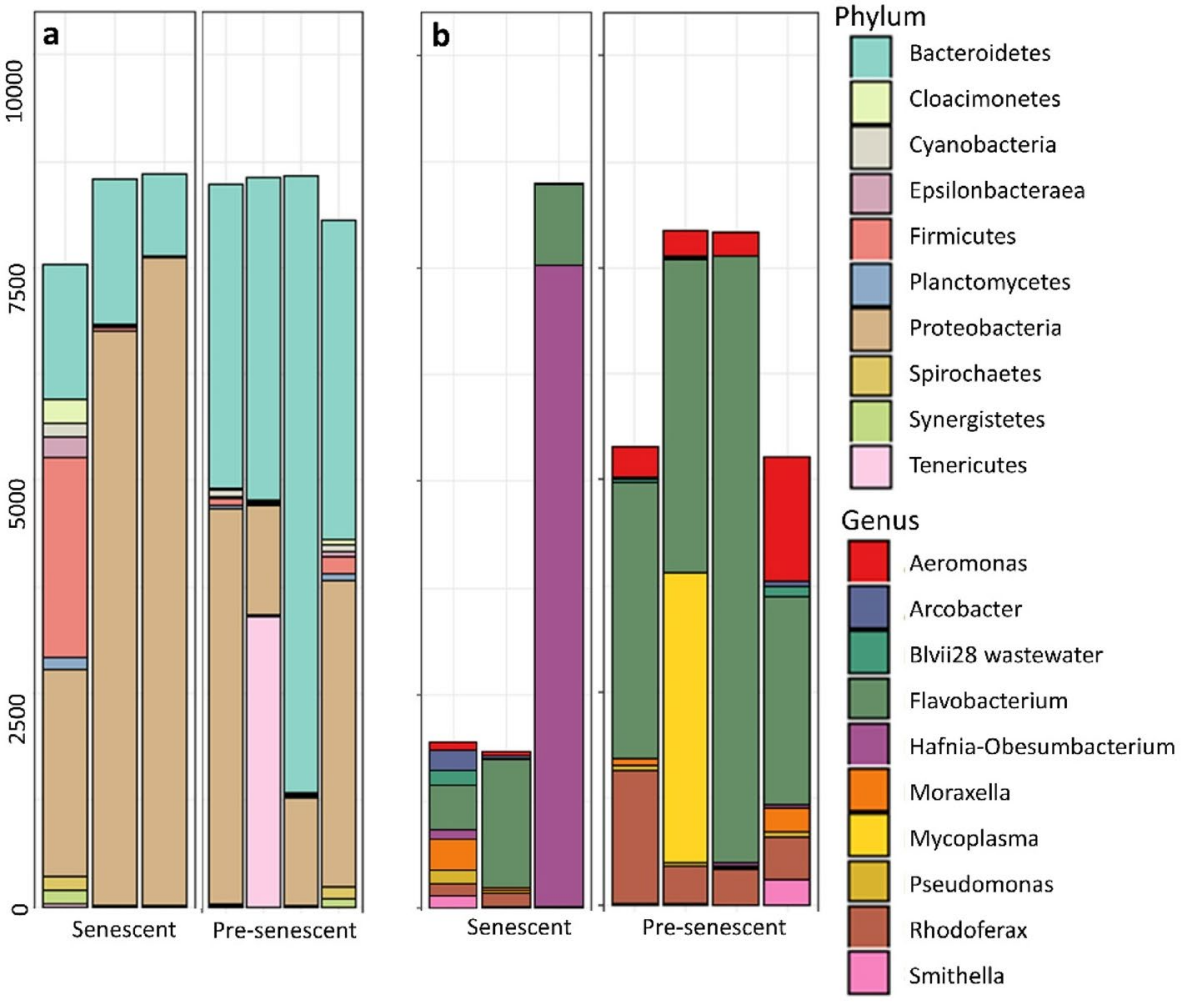
Rarefied abundances of (**a**) the top ten most abundant phyla and (**b**) the top ten most abundant genera in the guts of adult Chinook salmon following euthanasia for artificial spawning. Pre-senescent refers to individuals with relatively intact intestinal epithelia, and senescent refers to individuals with highly degraded intestinal epithelia.

A convergent NMDS solution for the adult samples was found after 49 iterations. Stress was nearly zero likely due to the smaller number of ASVs and samples in this dataset, but the NMDS plot revealed separation between adults with low versus high intestinal epithelial loss. A separate NMDS was generated for juvenile and adult samples together after subsampling to the minimum read depth for adults to ensure the two datasets were comparable. For this NMDS analysis, a solution was reached after 28 iterations, with a stress of 0.1. Although our very small sample size for adults precluded formal multivariate analysis comparing adults and juveniles, visualization along two NMDS dimensions demonstrated that all adults were more similar to moribund juveniles than surviving juveniles, and that senescent adults exhibited a shift that was dimensionally similar but more extreme than either moribund juveniles or pre-senescent adults (Fig. 4). Although we did not conduct formal statistical comparisons between senescent and pre-senescent adults due to small sample size that could lead to spurious conclusions, relative abundances of the top ten most abundant phyla and genera are shown in Fig. 5, and richness and diversity of senescent and pre-senescent adults are shown in Fig. S7.

## Discussion

In this work, we explored the effects of chronic corticosteroid elevation on the gut microbiome of Chinook salmon to assess the possible effects of life history-associated changes in cortisol that occur naturally throughout the lifetime of the host. We found that exogenous corticosteroids resulted in a significantly altered gut microbiome communities after three weeks of treatment. Gut microbiome variation three weeks post treatment was associated with subsequent morbidity among corticosteroid-treated fish. We also identified specific bacterial genera associated with corticosteroid treatment, morbidity, or both. We compared data from the experimental juveniles with samples from adult fish collected from a hatchery at various stages of senescence and found that senescent fish were more similar to moribund treated juveniles than control or surviving juveniles, suggesting a common dysbiotic signature of corticosteroid elevation regardless of life stage.

Our finding that corticosteroid treatments affect gut microbiome diversity and composition is consistent with studies in other organisms. Previous studies showed that cortisol elevation due to acute stressors associates with altered diversity and composition of the gut and skin microbiome in Atlantic salmon (*Salmo salar*)^16,18^. Specific environmental stressors, including thermal stress^55,56^ and crowding stress^57^ modulate the microbiota of other fish species. Chronic administration of corticosteroids has been shown to affect the mammalian gut microbiome^14^, and chronic stress-induced corticosteroid elevation associates with altered gut microbial communities in several mammals including grey squirrels^58^, and human children^59^. To our knowledge, however, this study is the first to directly assess the effects of chronic corticosteroid elevation on the gut microbiome of a fish host. We found that slow-release implants of cortisol or dexamethasone significantly altered microbiome composition. Additionally, we found differing effects of dexamethasone and cortisol on alpha diversity. Dexamethasone resulted in decreased alpha diversity, while cortisol resulted in increased alpha diversity. These findings are consistent with other studies, as stress-induced cortisol associated with increased alpha diversity in Atlantic salmon^16^, and dexamethasone treatment led to reduced alpha diversity in mice^60^. However, to our knowledge, this is the first study to simultaneously assess the effects of cortisol and dexamethasone on the gut microbiome. Although cortisol and dexamethasone have broadly similar effects on innate immunity^61^, their differential effects on gut microbial alpha diversity could be caused by differences in receptor specificity or potency of the two compounds. Cortisol binds to both mineralcorticoid and glucocorticoid receptors, whereas dexamethasone is almost totally specific to glucocorticoid receptors^62^. Additionally, dexamethasone is known to have higher glucocorticoid potency than cortisol in teleost fishes and other organisms^63^. These findings therefore highlight the need to study the mechanisms by which alternative corticosteroids can influence the gut microbiome.

We found that gut microbiome composition associated with subsequent morbidity in corticosteroid treated fish. Additionally, we found that the gut microbiomes of adult fish in various stages of senescence were more similar to moribund juveniles than to surviving juveniles. Though our study design did not allow us to assess causal relationships between microbiome variation and morbidity, we speculate that the microbiome shifts observed in fish that ultimately became moribund were related to corticosteroid-induced changes in immune function that led to increased susceptibility to opportunistic gill pathogens and ultimately to morbidity. In this regard, our findings align with previous studies in Atlantic salmon that identified associations between infectious disease and microbiome composition^19,30,64,65^. Bacterial gill disease, caused by a variety of opportunistic bacteria found in water, as well as infections by *Aeromonas salmonicida* and *Ichthyobodo nector*, are associated with fish that are immune compromised^66–68^. In particular, *I. nector* was observed in fish treated with dexamethasone^67^. We found opportunistic infections in treated fish in well water from a source that is typically free of pathogens^69^ as well as in untreated river water, suggesting that pathogens were present in the fish when they were obtained from the hatchery but did not proliferate until they were immunosuppressed with corticosteroids. Corticosteroids have long been known to play a role in regulating host immunity, particularly under conditions of stress^70^. In Pacific salmon, immune changes and increased pathogen susceptibility accompany the cortisol rises associated with major life history transitions, such as when young fish move from freshwater to saltwater^29^, and when adults return to spawn^71^. Prolonged stress, e.g. due to crowding, poor water quality, or thermal mismatch, can also result in chronically elevated cortisol and enhanced susceptibility to opportunistic pathogens^72–74^. Our findings demonstrate that these vulnerable life history stages and prolonged stress may also be accompanied by microbiome perturbation. Because the microbiome is known to play an important role in immune function and gut barrier integrity of vertebrates^75^, corticosteroid-induced changes to the microbiota may result in enhanced disease susceptibility, particularly if environmental conditions are not conducive to the re-assembly of a “healthy” microbiome following perturbation.

Although cortisol and dexamethasone had differing effects on microbiome diversity, we identified several bacterial genera that associated with both corticosteroid treatment types, suggesting a common signature of corticosteroid-induced dysbiosis in juvenile Chinook salmon. The bacterial genera that associated with both treatment types included *Candidatus Branchiomonas*, which includes a known agent of epitheliocysts in salmonids^76^, *Diaphorobacter*, which is a common fish gut microbe^77^ that is known to associate with immunosuppressive disorders in humans^78^, and *Peredibacter*, a genus that is associated with polymicrobial disease in Pacific oysters^79^. It is possible that these taxa represent opportunistic colonizers of immunosuppressed hosts. In addition to these biomarkers of corticosteroid treatment, we also identified taxa that associated with mortality after accounting for treatment, including *Diphorobacter* and *Fluviicola*. Because *Diphorobacter* increased in both corticosteroid treatment groups and also associated with mortality after accounting for treatment, this genus may represent a particularly sensitive biomarker of the host response to chronic glucocorticoid elevation. *Fluviicola* is a core microbe of zebrafish and found in other fish species, suggesting it may be a commensal bacterium that is important to fish physiology. However, similar to *Peredibacter*, this associates with polymicrobial disease in Pacific oysters. *Fluviicola* may therefore represent a common aquatic commensal that opportunistically proliferates in the context of host stress or immune dysfunction.

Altogether, our findings demonstrate that chronic corticosteroid elevation alters gut microbiome composition and diversity in Chinook salmon and may amplify potential pathogens. These findings have implications for Pacific salmon health and survival during life history events characterized by chronically elevated cortisol, including smolting, migration, and sexual maturation. Elucidating how corticosteroids interact with the gut microbiome could contribute to our understanding of the metabolic, osmoregulatory, and immune changes that accompany these life history transitions. In this work, we found that community-level and taxon-specific microbiome variation distinguished treated fish that became moribund from those that survived. These taxa could be causally related to morbidity, or they could indicate underlying physiological vulnerability to stress and disease. Regardless of causal directionality, these microbes could have potential utility for nonlethal monitoring of population health. Ultimately, understanding how the microbiome functions during periods of elevated corticosteroids could inform conservation and management interventions for Pacific salmon at vulnerable life history stages, in addition to clarifying our overall understanding of stress and the gut microbiome.

## Supplementary Information

Supplementary Information 1.

Supplementary Figures.

Supplementary Table S1.

## Data Availability

All 16S sequences and relevant ancillary data have been deposited in the NCBI Sequence Read Archive under accession number PRJNA881973.
