# Supplementary material for: Gut microbiome composition associates with corticosteroid treatment, morbidity, and senescence in Chinook salmon (Oncorhynchus tshawytscha)
Source: Sci Rep. 2023 Feb 13;13:2567. doi: 10.1038/s41598-023-29663-0 (PMC9925776; doi:10.1038/s41598-023-29663-0)
Supplement: Supplementary file 2 — Supplementary Figures. [file 41598_2023_29663_MOESM2_ESM.docx]

Supplementary Figures


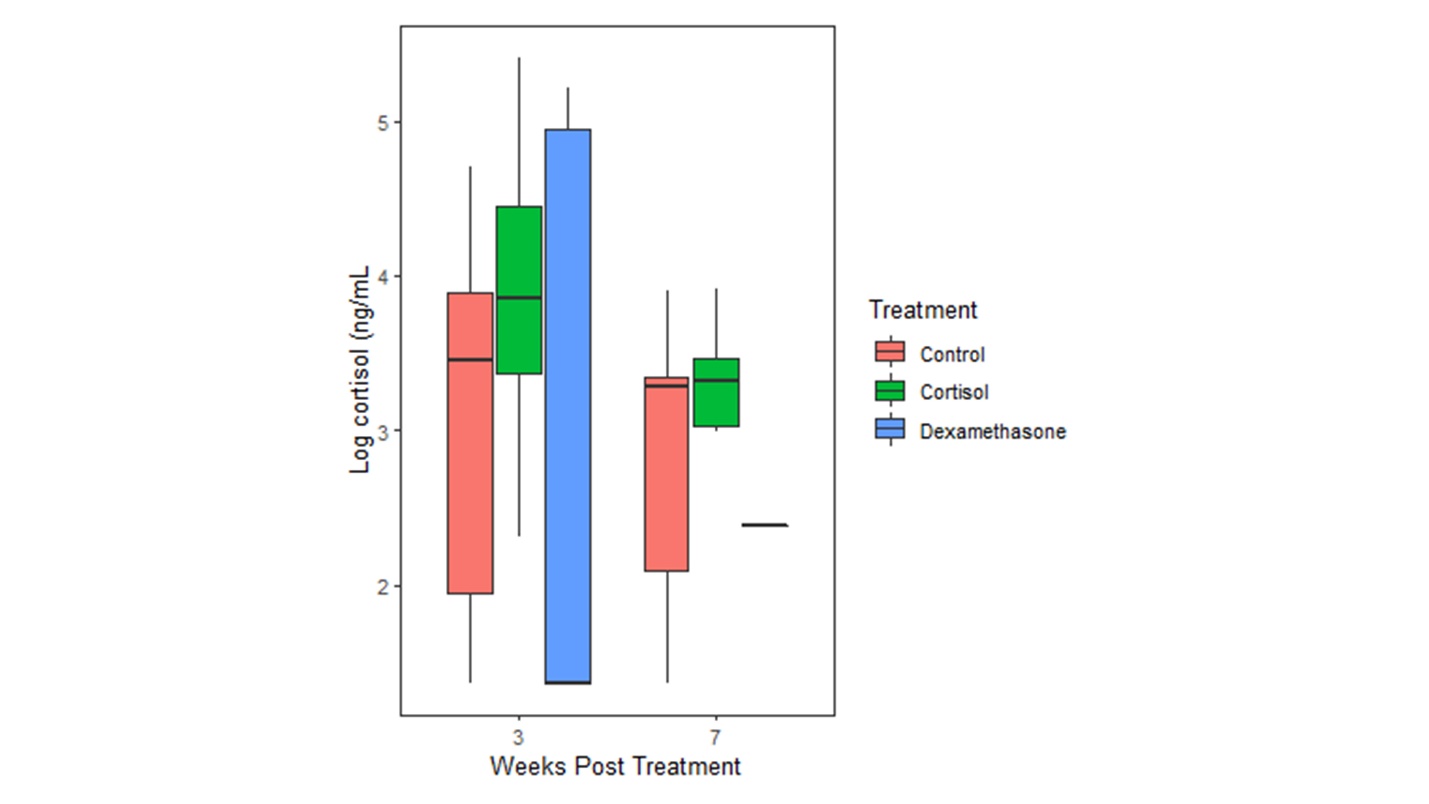


Figure S1: Log-transformed plasma cortisol concentration of juvenile Chinook salmon three and seven weeks after treatment with a slow-release cortisol or dexamethasone implant, or vehicle-only control.


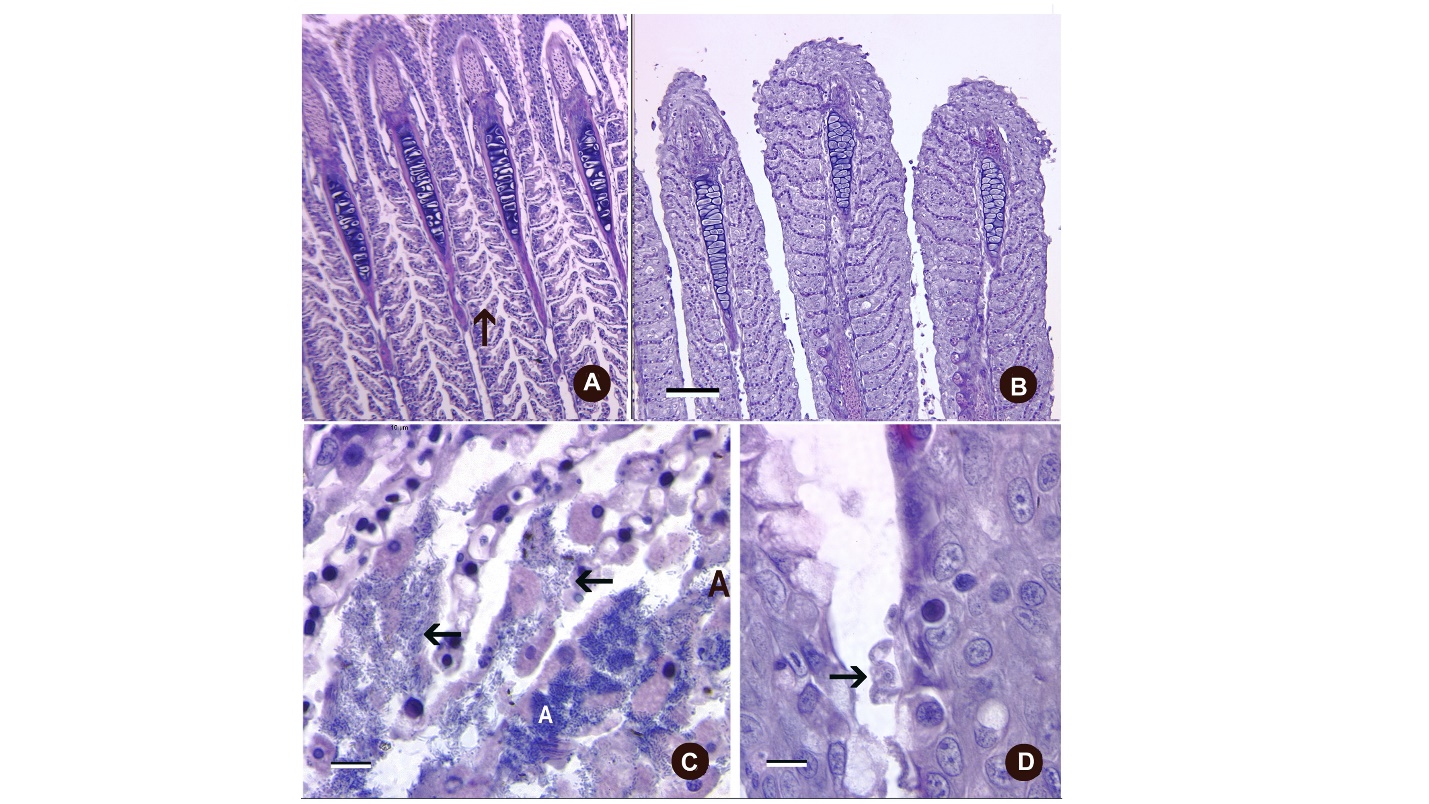


Figure S2: Gill histopathology (H&E) from representative (a) healthy and (b-d) moribund juvenile Chinook salmon. Fish that became moribund were characterized by gill hyperplasia and opportunistic gill infections. A. Normal, healthy gills showed minimal hyperplasia as evidenced by the space between secondary lamellae (arrow). Bar = 100 µm.  B. Gills from moribund fish showed severe, diffuse epithelia hyperplasia with fusion of lamellae. C. Affected gills were often infected with a mixture of bacteria (arrows)  and aggregates of bacteria (A) consistent with *Aeromonas salmonicida*. D. Some gills were infected with the protozoan flagellate *Ichthyobodo necator* (arrow)*.* Opportunistic pathogens such as these were likely the proximate cause of death in fish that were immunocompromised due to chronic corticosteroid elevation.


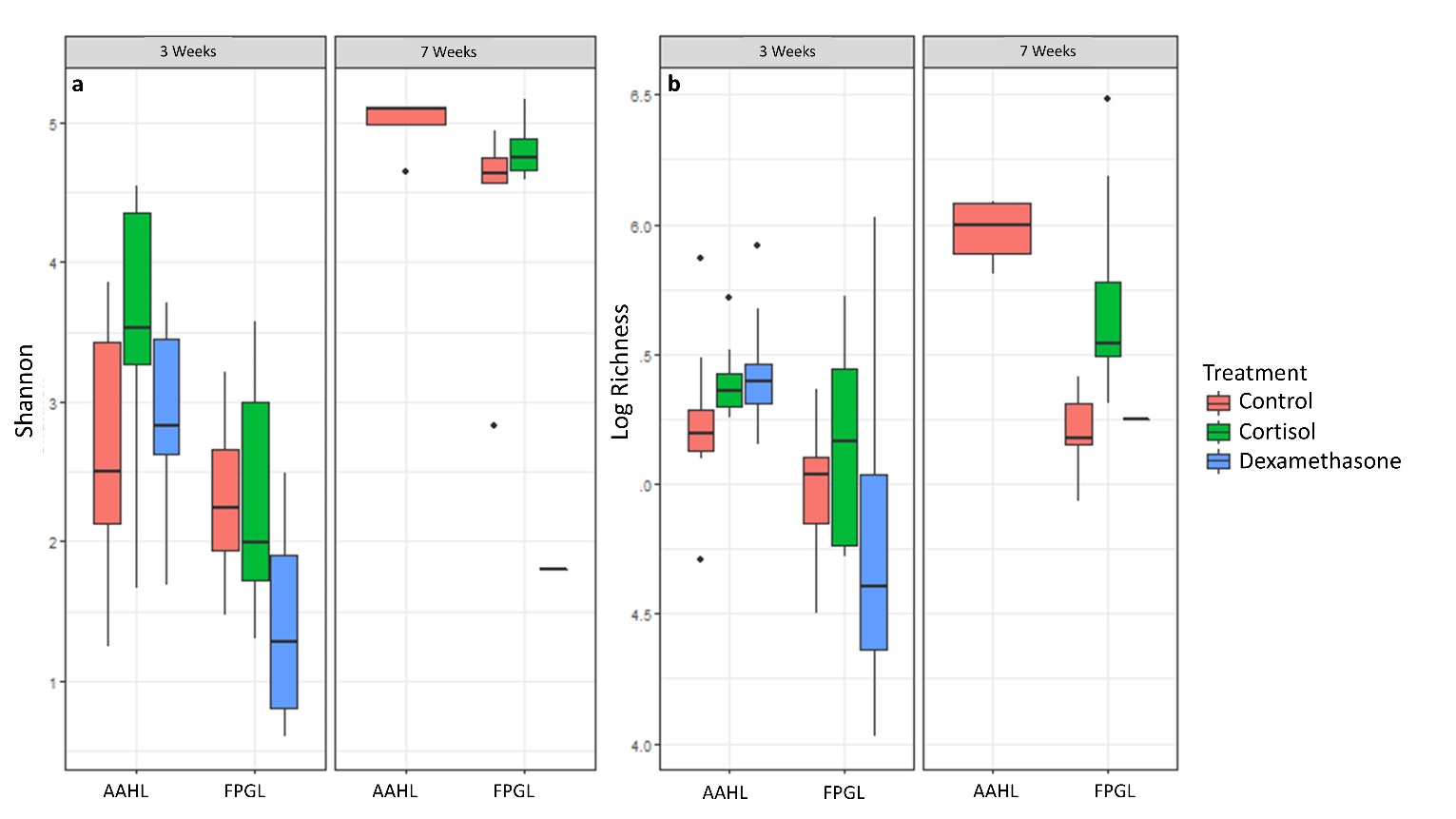


Figure S3: Shannon diversity (a) and log richness (b) of gut microbiomes of juvenile Chinook salmon three and seven weeks after treatment with a slow-release cortisol or dexamethasone implant, or vehicle-only control.


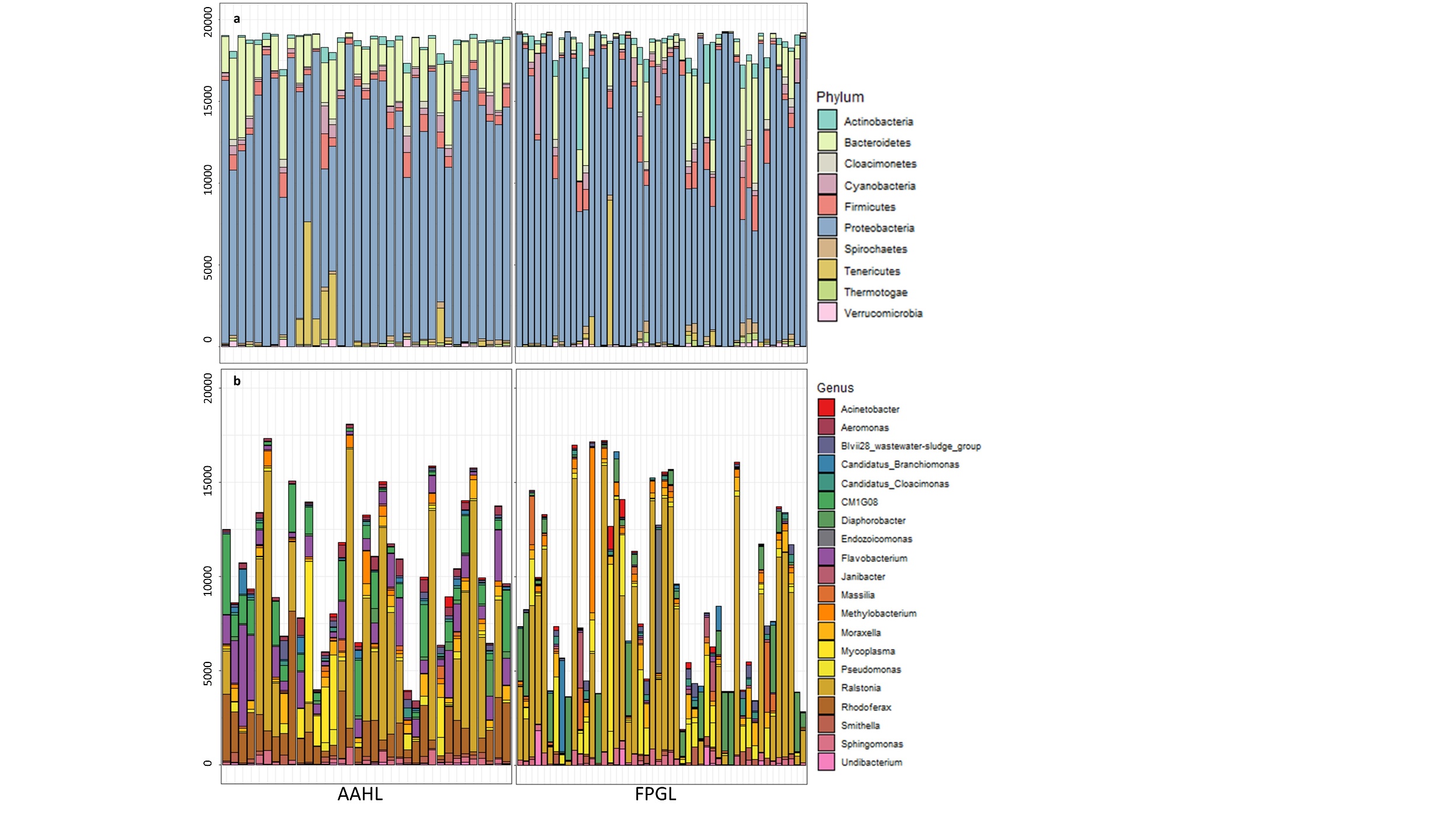


Figure S4: Abundances of (a) the top ten phyla and (b) the top ten genera in samples from fish in untreated river water (AAHL) versus well water (FPGL).


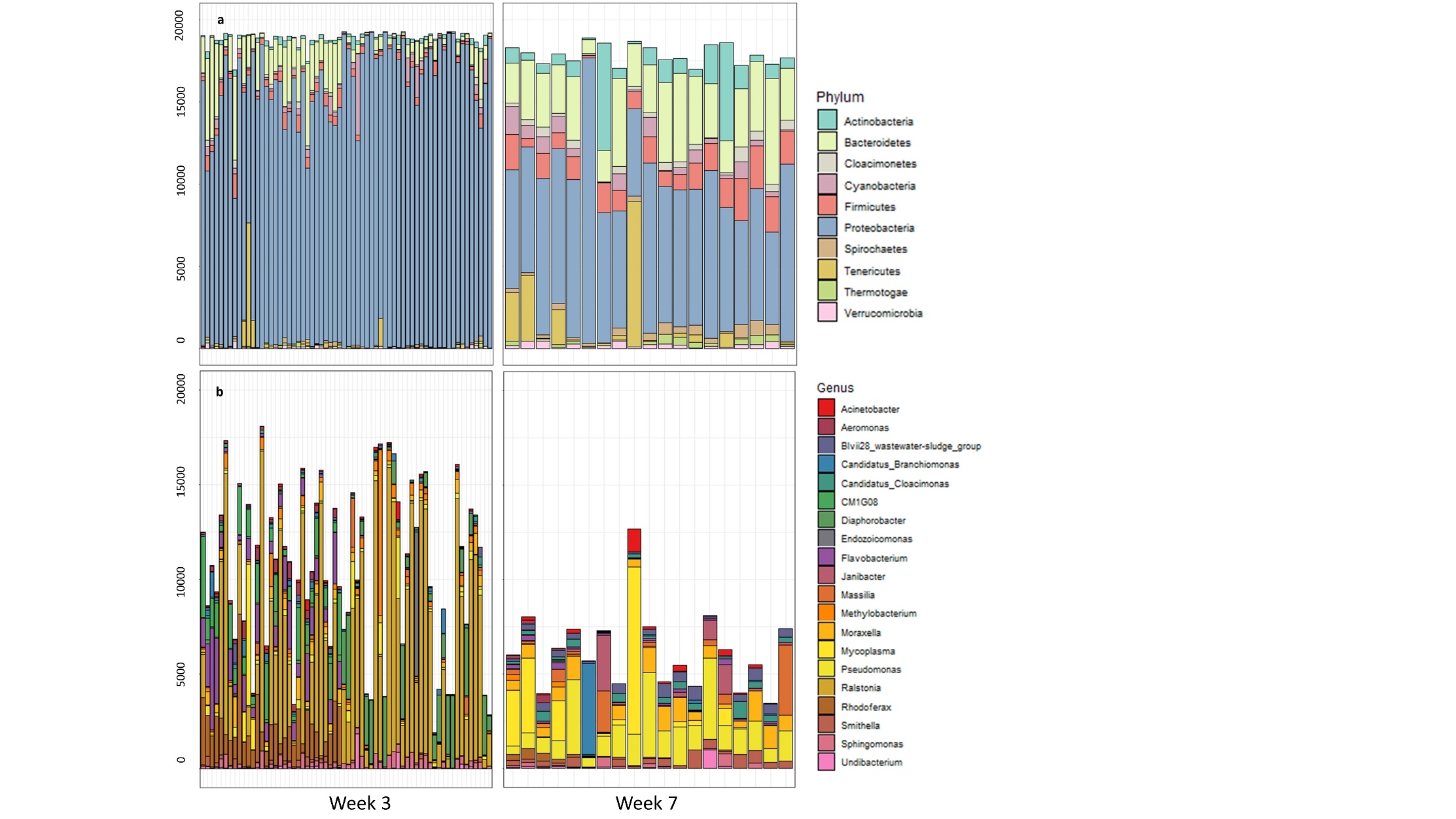


Figure S5: Abundances of (a) the top ten phyla and (b) the top ten genera in fish sampled after three weeks versus after seven weeks.


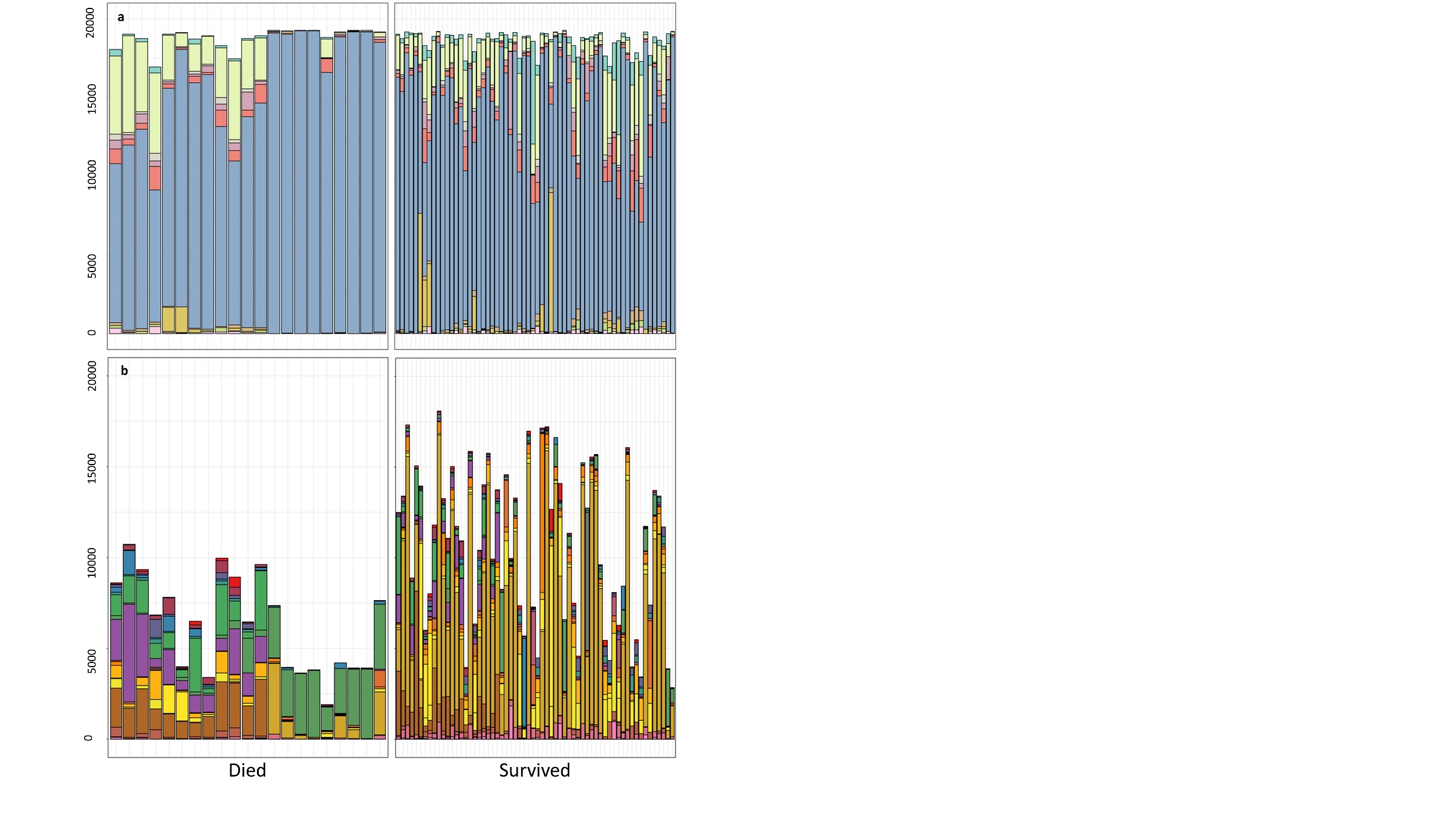


Figure S6: Relative abundances of (a) phyla and (b) genera of fish that became moribund after sampling versus those that did not.


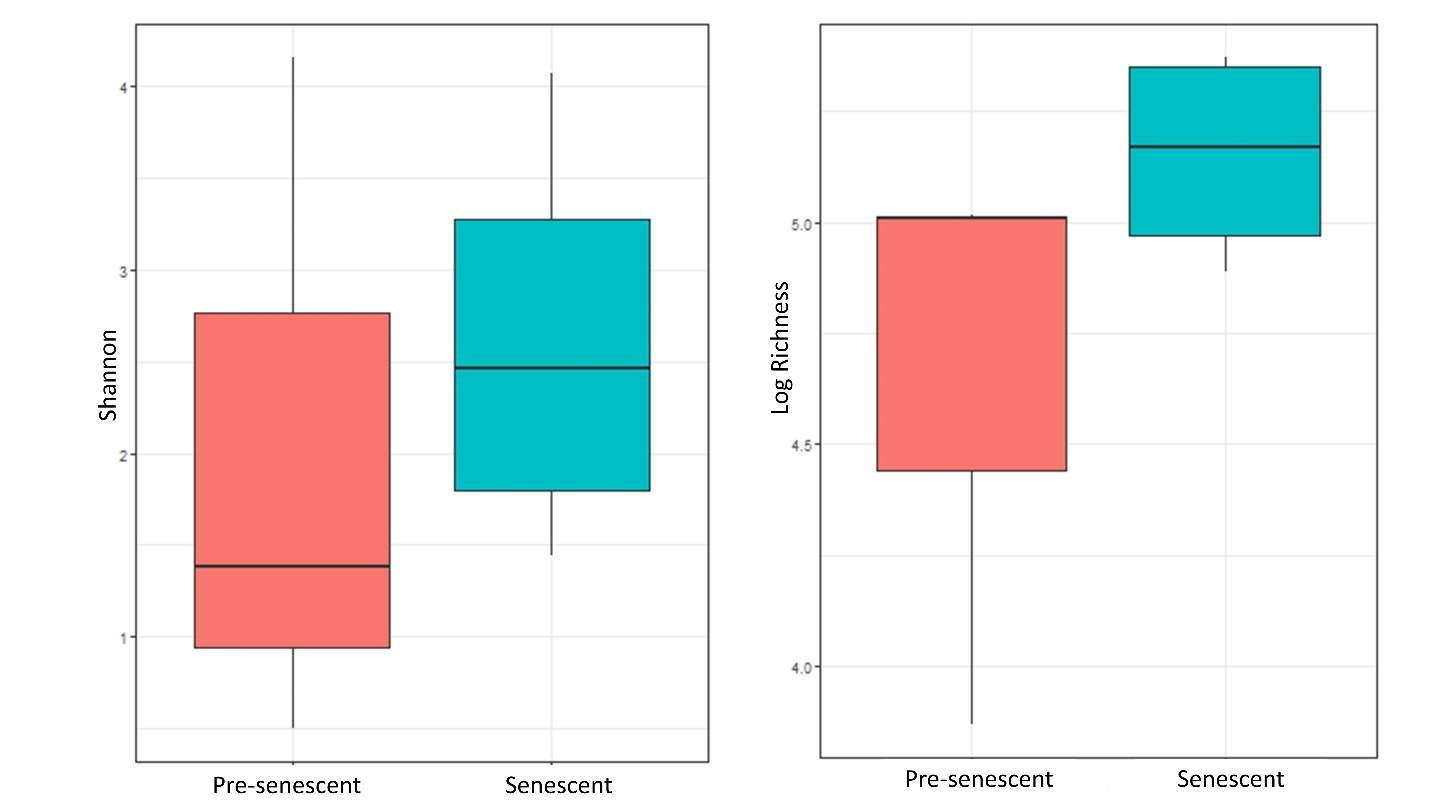


Figure S7: Shannon diversity and log richness of gut microbiota from adult Chinook salmon with highly degraded guts (senescent, n = 4) or relatively intact guts (pre-senescent, n = 3) intestinal epithelia.
