# Supplementary material for: Gut microbiome composition associates with corticosteroid treatment, morbidity, and senescence in Chinook salmon (Oncorhynchus tshawytscha)
Source: Sci Rep. 2023 Feb 13;13:2567. doi: 10.1038/s41598-023-29663-0 (PMC9925776; doi:10.1038/s41598-023-29663-0)
Supplement: Supplementary file 3 — Supplementary Table S1. [file 41598_2023_29663_MOESM3_ESM.docx]

| Table S1: The number of microbiome, histology, and cortisol samples collected at each time point. Mortalities include fish that died between the first and second sampling point. | | | |
| --- | --- | --- | --- |
| Sample Type | 3 weeks post treatment | Mortalities between 3-7 weeks | 7 weeks post treatment |
| Microbiome | 64 | 21 | 19 |
| Cortisol | 126 | NA | 70 |
